# Regenerative Endodontic Management of an Immature Necrotic Premolar Using Advanced Platelet-Rich Fibrin

**DOI:** 10.1155/2023/1135413

**Published:** 2023-01-31

**Authors:** Sepideh Hosseini, Nazanin Chitsaz, Mohammad Hassan Hamrah, Donya Maleki, Emad Taghizadeh

**Affiliations:** ^1^Department of Pediatric Dentistry, School of Dentistry, Guilan University of Medical Sciences, Rasht, Iran; ^2^Department of Endodontics, School of Dentistry, Tehran University of Medical Sciences, Tehran, Iran; ^3^Department of Public Health and Health Systems, Nagoya University Graduate School of Medicine, Nagoya, Japan; ^4^Faculty of Dentistry, Guilan University of Medical Sciences, Rasht, Iran; ^5^Department of Oral and Maxillofacial Surgery, School of Dentistry, Guilan University of Medical Sciences, Iran

## Abstract

Regenerative endodontic management is a feasible treatment for immature teeth with periapical radiolucency and necrotic pulp that simplifies continued root creation. Among the most prevalent health problems in an immature root is dental pulp necrosis, which is caused by caries, improper endodontic treatments, and trauma. Necrosis of the dental pulp can affect long-term tooth survival and preservation and serve as a source of bacteria infecting the periapical tissue and the maxillofacial space. Here, we report on the application of advanced platelet-rich fibrin plus (A-PRF+) therapy, as a regenerative endodontic treatment (RET), in a 12-year-old with necrotic pulp and asymptomatic apical periodontitis. Over a 24-month follow-up post-treatment, we observed resolving of symptoms and a complete root formation with considerable periapical healing.

## 1. Introduction

Pulp necrosis in immature teeth leads to the incomplete development of roots, consisting of thin walls and open apices, making endodontic management challenging [1]. The pulp necrosis etiology in immature permanent teeth can include trauma, caries, and the existence of dental anomalies including dens evaginatus or dens invaginatus [2].

Immature teeth with apical periodontitis and pulp necrosis make challenges for endodontic management owing to the incomplete development of roots, thin root walls, and open apices [1]. For these cases, the conventional treatment modality includes apexification with single-step mineral trioxide aggregate (MTA) or the application of calcium hydroxide (CaOH) paste as an apical barrier to obtain apex closure. These two approaches have the drawback of restricting normal physiological root development, which leads to thin and fragile roots [2]. A more recent therapeutic approach for such cases involves regenerative endodontic treatment (RET) in which root development is continued, conferring the advantages of vitality and maturation [3].

Several case studies have been published on immature necrotic teeth, indicating the potential of RET in stimulating the continuous formation of root length and width and preserving their structural integrity [4–6]. Using RET, healing of apical periodontitis is induced, restoring normal physiological functions of the pulp [7]. Continuous root development cannot be obtained with an apexification technique [8, 9].

More recently, studies have been performed on an autogenous material, platelet-rich fibrin (PRF), which serves as an osteoconductive scaffold with integrated growth factors for stimulating the cells for a regenerative response. While this technique has been utilized in dentistry for repairing different lesions and regeneration of dental and oral tissues, it can also be employed as a scaffold for pulp regeneration in an immature necrotic tooth [10].

The present case report describes the management of a non-vital immature premolar tooth causing symptomatic apical periodontitis and necrosis of the pulp.

## 2. Case Presentation

The patient was an 11-year-old boy referred to the Department of Endodontic Dentistry of Tehran University of Medical Science, Tehran, Iran, with a complaint of spontaneous pain in the lower left premolar (tooth #20).

**Figure 1:**
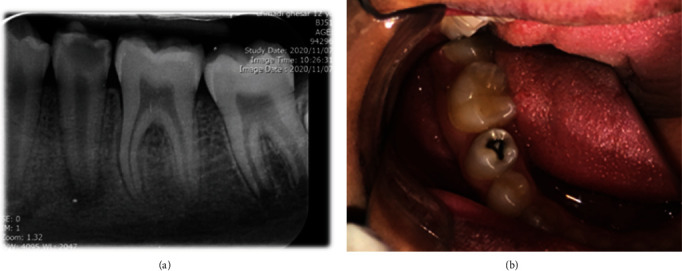
(a) The initial periapical radiograph showing prominent tubercles due to dens evaginatus, periapical radiolucency, and deep caries in tooth #20 with an immature apex; (b) preoperative photograph and caries in tooth #20; the intact tubercle can be observed on the occlusal surface of this tooth.

General dental, medical, and traumatic histories were obtained, and no systemic disease was noted. Extensive caries was revealed by clinical examination on the occlusal surface as well as sensitivity to palpation and percussion and dens evagination addressed as a differential diagnosis for this case. A negative response was obtained by the vitality test with electric pulp testing (EPT) (Digitest 3 Parkell, USA) and Endo-Frost cold spray (Roeko, Coltene Whaledent, Langenau, Germany), which represented no mobility. In the radiographic examination, an immature root was found along with deep caries, open apex, and periapical radiolucency. Additional caries was not found in the other teeth (Figure 1).

Necrotic pulp was diagnosed with symptomatic apical periodontitis. REP was proposed as the best feasible option for treatment. A full explanation was provided to the patient and his family about the risks and benefits of REP, and informed consent for the procedure was obtained.

An inferior alveolar nerve block was given with 2% lidocaine and 1 : 100,000 epinephrine (Daroupakhsh, Tehran, Iran) on the first visit. Caries removal and access cavity were conducted with diamond burs (Dentalree, USA) followed by rubber dam isolation. An apex locator (Woodpecker, Foshan, China) along with radiography (XGenus, De Götzen SRL, Varese, Italy) was used to verify the working length. To irrigate the canals, 20 ml of 1.5% sodium hypochlorite (NaOCl) with ultrasonic passive activation was used. This was followed by irrigation with normal saline and drying by paper point (Dentplus, Choonchong, Korea). After applying CaOH into the canal, the access cavity was sealed temporarily with RMGI (Fuji IX, GC Corporation, Tokyo, Japan).

**Figure 2:**
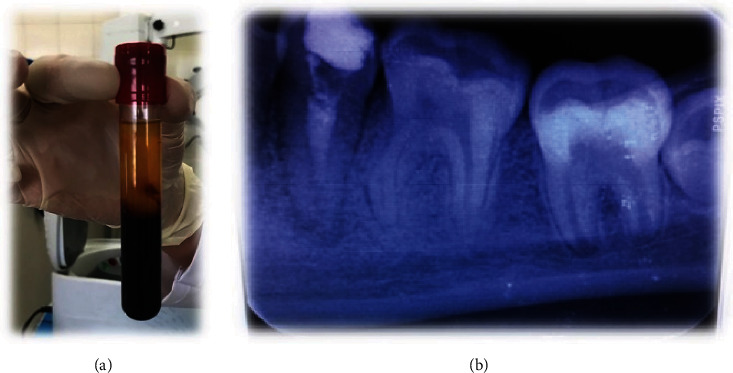
(a) Preparation of A-PRF+; (b) placing MTA on A-PRF+ and a temporary filling.

The second session was appointed after 3 weeks. The patient did not have any complications. Using 3% mepivacaine, the tooth was anesthetized without a vasoconstrictor (Daroupakhsh, Tehran, Iran). The temporary restorative material was removed after isolation with a rubber dam. Normal saline was used to wash Triple antibiotic paste (TAP) out of the canal. The canal was irrigated thoroughly with 20 ml of 17% Ethylenediamide tetraacetic acid (EDTA). PRF was prepared by drawing 10 ml of whole blood and centrifugation at 1300 rpm for 8 minutes. After drying the canals, a 40-K file was inserted 2 mm beyond the apex in the canal to initiate bleeding (Dentsply Maillefer, Ballaigues, Switzerland). The advanced platelet-rich fibrin plus (A-PRF+) was injected into the root canal to a level of 3 mm under the cementoenamel junction (CEJ). To reduce the discoloration effect of MTA, a dentin bonding agent was placed on the dentinal walls. Ortho MTA (MTA-Ang; A Angelus, Londrina, PR, Brazil) was placed as a coronal barrier. A moist cotton pellet was placed over the MTA in the pulp chamber, and the access cavity was temporized again with GC1 filling (Figure 2).

A week later, during the third appointment, the glass ionomer and cotton pellet were removed. By setting MTA, restoration of the tooth was performed with composite (Clearfil AP-X, Kuraray Medical, Tokyo, Japan).

**Figure 3:**
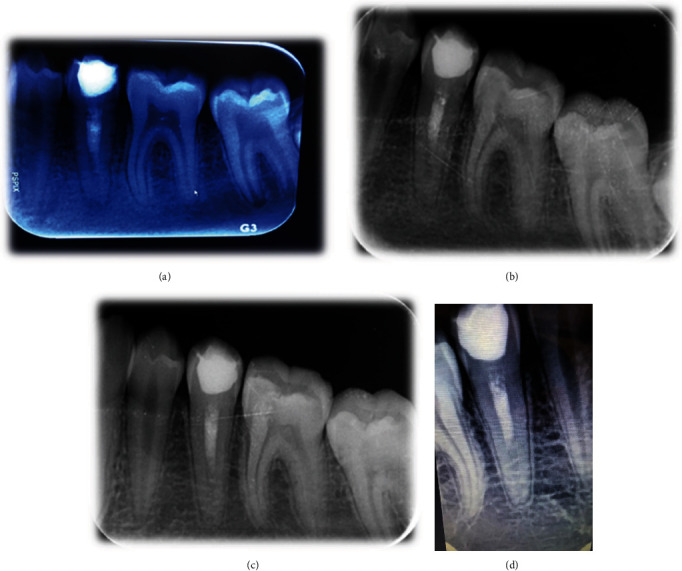
The periapical radiograph during follow-ups at months 3 (a), 6 (b), 12 (c), and 24 (d).

**Figure 4:**
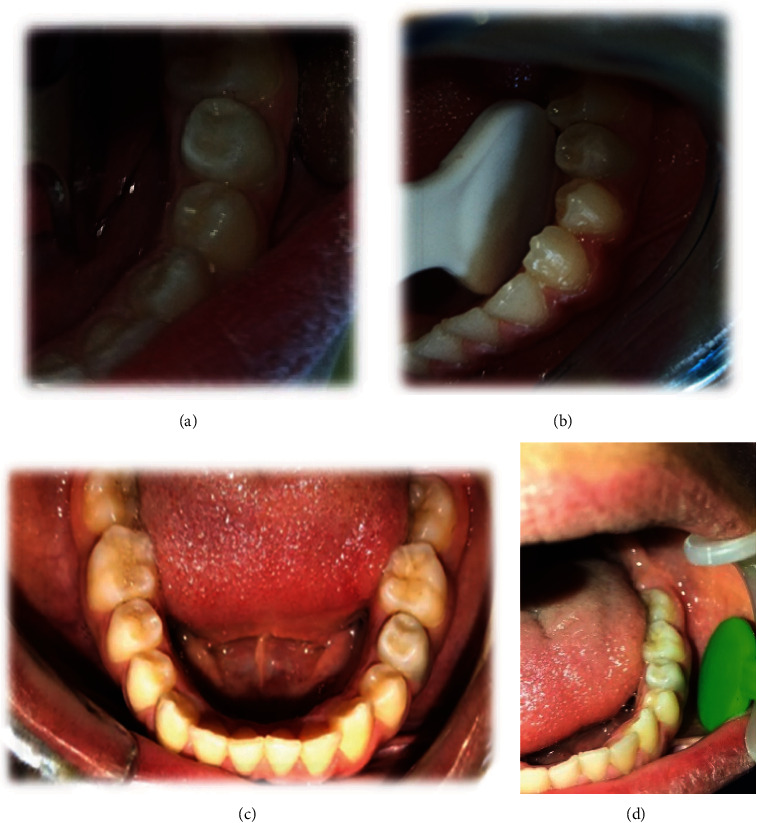
The postoperative photograph during follow-ups at months 3 (a), 6 (b), 12 (c), and 24 (d).

During follow-ups at months 3, 6, 12, and 24, the tooth remained asymptomatic with no sign of resorption, clinically or radiographically. Healing of the periapical lesion was illustrated through a radiographic assessment, which showed incremented root thickness, length, and width, indicating the apical closure (Figures 3 and 4).

## 3. Discussion

This report described the administration of RET on immature pulp necrosis of a permanent premolar tooth with periapical radiolucency. The process conducted for this subject involved the placement of a freshly prepared PRF+ membrane inside the canal. From clinical experience, incremental placement of PRF fragments is more convenient compared to the placement of a membrane [11]. An MTA was directly placed over PRF to achieve a coronal seal [12, 13]. Satisfactory radiographic and clinical results were obtained over a 24-month follow-up.

Apexification and RET are two treatment modalities for an immature tooth with pulp necrosis. Apexification requires the intracanal application of CaOH, which should be replaced every 3 months [14, 15]. The long-term application of an intracanal medication and the development of defects in the root walls owing to the CaOH's porous attributes increase the possibility of root fracture. On the other hand, RET, a shorter treatment, increases the root thickness and length and results in complete root development in a brief period [16–18]. MTA has been extensively used in the last decade for the apexification of non-vital immature teeth. Although it has several setbacks such as tooth discoloration and weakening of the dentin wall, it reduces the treatment time and is more effective than CaOH [19], and therefore, it can be considered as a treatment option to save the tooth. In this case, the American Association of Endodontists (AAE) recommendations were followed for irrigation protocols and the application of CaOH medication inside the root canal for 3 weeks [20].

Platelet products have great potential in regenerative medicine owing to their potency in releasing and storing biologically active materials, regulating the innate immune response, and combatting infection [21]. PRF has been presented as an autogenous source of blood growth factors, serving as a tool for tissue regeneration in modern medicine [22]. It was introduced in 2001 [23], and since then it has been utilized extensively in dentistry for various procedures, with demonstrated effectiveness in treating gingival recessions, extraction socket management, intrabody defect regeneration, and sinus elevation [24–26]. One of its main advantages is the obtaining of immune-compatible growth factors at a low cost without the use of anti-coagulants [17]. Moreover, PRF has been associated with superior results in the regeneration of the pulp-dentin complex in endodontic treatments [10]. Reducing the centrifugation g-force is shown to increase the total leukocyte numbers within PRF matrix scaffolds, a modification known as A-PRF+ [27]. According to Fujioka's study, growth factors are released at significantly higher levels by A-PRF+ compared to L-PRF or A-PRF [22]. The decision on the use of A-PRF+ for RET in the present case was made based on the above information.

According to a meta-analysis, the mean success rate after the first year for apical closure or reduction has been 85% for both PRP (85.1%) and PRF (85.2%). The same value for root lengthening was 64.2% (PRP) and 74.1.2% (PRF). The periapical lesion healing response as well as dentinal wall thickening rates was 100% for both PRP and PRF [28]. However, Kobayashi et al. examined the release of various growth factors from PRP, PRF, and A-PRF+ for 15 minutes, 59 minutes, 8 hours, 1 day, 3 days, and 10 days and found that A-PRF+ released the maximum amount of growth factors over a longer duration when compared to PRF or PRP, which would be beneficial for regenerative procedures [29]. Moreover, other studies conducted on immature necrotic teeth have demonstrated the potential of RET in stimulating continuous root length and width formation and preserving their structural integrity [4–6]. The most important advantage of RET is, therefore, the continued root development, which cannot be obtained using an apexification technique [8, 9].

The tooth was deemed clinically asymptomatic, and there was a complete root formation with considerable periapical healing radiographically and a successful outcome. In addition to the limitation of case report in term of its essence, it should be mentioned that it was not possible to confirm the vitality of the pulp after regeneration due to the lack of access to laser Doppler flowmetry and pulse oximeter.

## 4. Conclusion

Regenerative endodontic treatment with A-PRF+ was effective in the treatment of apical periodontitis and immature permanent premolar with pulp necrosis. For 24 months, follow-up appointments were conducted, in which the tooth was deemed clinically asymptomatic with a complete root formation and considerable periapical healing, demonstrated radiographically.

## Data Availability

Data supporting this research article are available from the corresponding author or first author on reasonable request.
